# Immunogenicity and transmission-blocking potential of quiescin sulfhydryl oxidase in *Plasmodium vivax*

**DOI:** 10.3389/fcimb.2024.1451063

**Published:** 2024-08-27

**Authors:** Wenqi Zheng, Shitong Cheng, Fei Liu, Xinxin Yu, Yan Zhao, Fan Yang, Sataporn Thongpoon, Wanlapa Roobsoong, Jetsumon Sattabongkot, Enjie Luo, Liwang Cui, Yaming Cao

**Affiliations:** ^1^ Department of Immunology, College of Basic Medical Sciences, China Medical University, Shenyang, China; ^2^ Department of Clinical Laboratory Medicine, Affiliated Hospital of Inner Mongolian Medical University, Hohhot, China; ^3^ National Clinical Research Center for Laboratory Medicine, Department of Laboratory Medicine, The First Hospital of China Medical University, Shenyang, China; ^4^ Mahidol Vivax Research Unit, Faculty of Tropical Medicine, Mahidol University, Salaya, Thailand; ^5^ Department of Pathogen Biology, College of Basic Medical Sciences, China Medical University, Shenyang, China; ^6^ Department of Internal Medicine, Morsani College of Medicine, University of South Florida, Tampa, FL, United States

**Keywords:** *Plasmodium vivax*, quiescin sulfhydryl oxidase, transmission-blocking vaccine, transgenic parasite, DMFAS

## Abstract

**Background:**

Transmission-blocking vaccines (TBVs) can effectively prevent the community’s spread of malaria by targeting the antigens of mosquito sexual stage parasites. At present, only a few candidate antigens have demonstrated transmission-blocking activity (TBA) potential in *P. vivax*. Quiescin-sulfhydryl oxidase (QSOX) is a sexual stage protein in the rodent malaria parasite *Plasmodium berghei* and is associated with a critical role in protein folding by introducing disulfides into unfolded reduced proteins. Here, we reported the immunogenicity and transmission-blocking potency of the PvQSOX in *P. vivax*.

**Methods and findings:**

The full-length recombinant PvQSOX protein (rPvQSOX) was expressed in the *Escherichia coli* expression system. The anti-rPvQSOX antibodies were generated following immunization with the rPvQSOX in rabbits. A parasite integration of the *pvqsox* gene into the *P. berghei pbqsox* gene knockout genome was developed to express full-length PvQSOX protein in *P. berghei* (Pv-Tr-PbQSOX). In western blot, the anti-rPvQSOX antibodies recognized the native PvQSOX protein expressed in transgenic *P. berghei* gametocyte and ookinete. In indirect immunofluorescence assays, the fluorescence signal was detected in the sexual stages, including gametocyte, gamete, zygote, and ookinete. Anti-rPvQSOX IgGs obviously inhibited the ookinetes and oocysts development both *in vivo* and *in vitro* using transgenic parasites. Direct membrane feeding assays of anti-rPvQSOX antibodies were conducted using four field *P. vivax* isolates (named isolates #1–4) in Thailand. Oocyst density in mosquitoes was significantly reduced by 32.00, 85.96, 43.52, and 66.03% with rabbit anti-rPvQSOX antibodies, respectively. The anti-rPvQSOX antibodies also showed a modest reduction of infection prevalence by 15, 15, 20, and 22.22%, respectively, as compared to the control, while the effect was insignificant. The variation in the DMFA results may be unrelated to the genetic polymorphisms. Compared to the *P.vivax* Salvador (Sal) I strain sequences, the *pvqsox* in isolate #1 showed no amino acid substitution, whereas isolates #2, #3, and #4 all had the M361I substitution.

**Conclusions:**

Our results suggest that PvQSOX could serve as a potential *P. vivax* TBVs candidate, which warrants further evaluation and optimization.

## Introduction

Malaria remains a significant public health problem in tropical and subtropical regions, with an estimated 249 million malaria cases and 608,000 deaths in 2022 (WHO, 2023). Among the five *Plasmodium* species causing malaria in humans, *P. falciparum* or *P. vivax* are the primary culprits, with *P. falciparum* accounting for the majority of morbidity and mortality, particularly in Africa. *P. vivax* has a broader geographical distribution and is the predominant malaria parasite species outside Africa (Baird, 2013; de Jong et al., 2020). Over the past decade, *P. vivax* malaria has contributed to 6–17 million malaria cases each year, with a substantial proportion occurring in Asia and America (WHO, 2023). Compared to other *Plasmodium* species, *P. vivax* exhibits high transmission potential due to its early production of gametocytes and enhanced infectivity to mosquitoes (Mueller et al., 2009). In addition, its ability to relapse from dormant hypnozoites significantly extends the period of transmission after the initial infection (Mueller et al., 2009). Therefore, novel interventions are urgently needed to control and eliminate *P. vivax* malaria.

Transmission-blocking vaccines (TBVs) represent a promising strategy for malaria elimination. TBVs aim to disrupt malaria transmission by eliciting antibody-mediated responses against *Plasmodium* proteins expressed during the sexual and early sporogonic stages, thereby interfering with parasite development in the mosquito vector (malERA Consultative Group on Vaccines, 2011). While TBVs do not directly protect vaccinated individuals from disease, they have the potential to shield communities from malaria transmission (Kilama and Ntoumi, 2009). In addition, TBVs may also slow down the transmission of drug- and vaccine-resistant strains and prolong the life span of existing antimalarial drugs and vaccines. Mathematical models have underscored the utility of TBVs in malaria elimination (Eckhoff, 2013). In recent years, several TBV candidate antigens of *P. vivax* have been identified and characterized, including gametocyte antigens Pvs48/45 and Pvs230, and the ookinete antigen Pvs25. Recombinant Pvs48/45 in monkey and mouse models demonstrated complete inhibition of parasite transmission to mosquitoes in artificial membrane-feeding assays (Arevalo-Herrera et al., 2015). Cysteine-rich domains I–IV of Pvs230 delivered by DNA vaccination also elicited functional antibody responses in mice, and the TBV activity was complement-independent (Tachibana et al., 2012). As the best-characterized *P. vivax* TBV candidate, Pvs25 expressed in yeast completely blocked *P. vivax* transmission to mosquitoes (Hisaeda et al., 2000). Furthermore, recombinant Pvs25 protein expressed in yeast and formulated in Alhydrogel^®^ also induced antibodies with significant transmission-blocking activity (TBA) in Phase I trials (Malkin et al., 2005), although a subsequent trial was halted due to reactogenicity (Wu et al., 2008). Therefore, new and effective TBV candidates for *P. vivax* remain to be discovered.

Quiescin sulfhydryl oxidase (QSOX), responsible for catalyzing the insertion of disulfide bonds into unfolded proteins, has garnered attention for its potential as a TBV candidate. It is a conserved enzyme in *Plasmodium* species and is expressed on the surface of gametes and ookinetes in *P. berghei* (Zheng et al., 2020). While QSOX has demonstrated efficacy in blocking transmission in rodent malaria models, its transmission-blocking potential in *P. vivax* remains unexplored. This study aims to characterize the immunogenicity and transmission-blocking potential of PvQSOX (PVX_101000), the QSOX ortholog in *P. vivax*. Our findings indicate that anti-rPvQSOX antibodies generated after immunization with the recombinant PvQSOX protein recognized native PvQSOX and exhibited TBA against transgenic *P. berghei* parasites and *P. vivax* field isolates. These results underscore the potential of PvQSOX as a viable candidate for *P. vivax* TBV development.

## Materials and methods

### Maintenance of parasites, mosquitoes, and mice

Female 6- to 8-week-old BALB/c mice and female New Zealand White rabbits (Beijing Animal Institute, Beijing, China) were used for animal experiments. *P. berghei* (ANKA strain 2.34) was maintained in mice by serial passage. Adult (3–4 days old) *Anopheles dirus* and *An. stephensi* mosquitoes were maintained in an insectary at 25°C ± 1°C, 80% relative humidity, and 12 h light and dark cycle and fed with 10% (w/v) glucose solution. Animal experiments were carried out following the guidelines of the Animal Ethics Committee of China Medical University.

### Cloning, expression, and purification of recombinant PvQSOX

For the expression of recombinant PvQSOX (rPvQSOX), the *pvqsox* gene encoding amino acids (aa) 40–553 of PvQSOX of the Salvador (Sal) I-I strain without the predicted signal peptide was harmonized for production in *Escherichia coli* using the codon harmonization algorithm in the EuGene software v0.92 (Gaspar et al., 2012). The *pvqsox* fragment (121–1662 bp) was synthesized (GenScript Biotech Corporation, Nanjing, China) and cloned into a pET30a (+) vector (Life Technologies, Carlsbad, CA) with a C-terminal 6 × His tag. The rPvQSOX was expressed in *Escherichia coli* BL-21 (DH3) cells. Cells were grown at 37°C to an optical density (OD) of ~0.5 and induced with 1 mM isopropyl-β-D-thiogalactopyranoside (IPTG) at 20°C for 12 h. The culture was harvested by centrifugation at 6,000 ×g for 10 min at 4°C, and the pellet was re-suspended in a solubilization buffer containing 6 M guanidine-HCl, 50 mM KH_2_PO_4_, 50 mM K_2_HPO_4_, 150 mM NaCl, and shaken for 1 h at room temperature. The solubilized inclusion body was centrifuged at 12,000 ×g for 30 min at 4°C, and the supernatant was collected. The proteins were purified with Ni-NTA agarose column (Qiagen, Venlo, Netherlands) as described by the manufacturer. Briefly, the column was equilibrated with the denaturing binding buffer (8 M urea, 20 mM sodium phosphate, 500 mM NaCl, pH 7.8), then washed two times with wash buffer 1 (8 M urea, 20 mM sodium phosphate, 500 mM NaCl, pH 6.0) followed by three washes with wash buffer 2 (8 M urea, 20 mM sodium phosphate, 500 mM NaCl, pH 5.3). Finally, the rPvQSOX protein was eluted in the elution buffer (8 M urea, 20 mM sodium phosphate, 500 mM NaCl, pH 4).

Refolding was done by dialysis in descending denaturant concentrations with modifications (Maeda et al., 1995; Malekian et al., 2019). First, proteins were dialyzed against a refolding buffer (50 mM Tris, 2 M Urea, pH 8) at 4°C overnight to remove urea slowly. Then, the denatured rPvQSOX proteins were refolded in a buffer containing 50 mM Tris, 250 mM NaCl, 50 mM arginine, 1 mM reduced glutathione, 0.1 mM oxidized glutathione, pH 8.0, and dialyzed overnight at 4°C. The proteins were concentrated using Amicon Ultra 3kD centrifugal filters (Millipore, Billerica, MA). The protein solution was mixed with 5% SDS buffer either containing 2% β-mercaptoethanol (reducing conditions) or without β-mercaptoethanol (non-reducing conditions) separated by 10% SDS-PAGE, and stained with Coomassie brilliant blue.

As the negative control for immunization, the recombinant glutathione S-transferase (rGST) protein was expressed from the vector pGEX-4T-1 (+) in *Escherichia coli* BL-21 (DH3) as previously described (Qiu et al., 2020; Zheng et al., 2020).

### Generation of anti-rPvQSOX polyclonal antibodies

Rabbits (n = 3) were subcutaneously immunized with 250 μg rPvQSOX emulsified in Freund’s adjuvant. Three booster immunizations of 250 μg rPvQSOX emulsified in incomplete Freund’s adjuvant were done at weeks 2, 5, and 8. As negative controls, rGST was administered using the same route and schedule. Ten days after the last immunization, the immune sera were collected and then pooled for each group. The polyclonal antibodies (IgGs) were purified from immune sera using Protein A columns. The BCA Protein Assay Kit (TaKaRa, Japan) was used to determine the concentrations of antibodies against rGST and rPvQSOX. For mosquito feeding assays, the IgGs were adjusted to 12 mg/ml using PBS according to the antibody concentrations.

### Enzyme-linked immunosorbent assay

Enzyme-linked immunosorbent assay (ELISA) was performed as previously described (Zheng et al., 2020). Briefly, 96-well ELISA plates (Nunc F96; Fisher Scientific) were incubated overnight at 4°C with purified rPvQSOX (5 µg/ml) in 0.15 M PBS (pH 7.2). After washes with PBS-T (0.05% Tween 20 in 0.1 M PBS, pH 7.4), plates were blocked with the blocking buffer [PBS-T, 3% bovine serum albumin (BSA), pH 7.4] for 2 h at room temperature. Then, the pooled rPvQSOX sera or rGST sera diluted from 1:1000 to 1:1024000 in the blocking buffer were added to each well and incubated at 37°C for 2 h. Plates were washed with PBS-T, and 0.1 ml HRP-conjugated anti-rabbit IgG (Invitrogen, Waltham, USA) diluted in 3% BSA in PBS-T was added to each well and incubated for 2 h at room temperature. After five final washes, tetramethylbenzidine (Amresco, Solon, USA) was added, and the reaction was stopped by 2 mM H_2_SO_4_. Plates were read at 490 nm using a SpectraMax 340 PC reader (Molecular Devices) and analyzed by GXP Validated SOFTmax Pro software. The IgG endpoint titers were read from the highest serum dilution greater than the cut-off value, defined as the mean plus 3 standard deviations OD reading of pooled rGST control sera.

### Generation of transgenic *P. berghei* expressing PvQSOX

Plasmids were designed to either knock out the endogenous *pbqsox* gene (Pb-KO line) or replace it with a *pvqsox* gene to obtain transgenic *P. berghei* expressing full-length PvQSOX protein in place of endogenous PbQSOX (Pv-Tr-PbQSOX line). To generate the knockout plasmid for double-crossover homologous recombination, an 858 bp upstream fragment containing the 5’ UTR and a 981 bp downstream fragment containing the 3’ UTR was obtained by PCR amplification using *P. berghei* genomic DNA as the template and primers 5’ UTR-F/5’ UTR-R and 3’ UTR-F/3’ UTR-R (**Supplementary Table S1**). The *pbqsox* 5’ UTR fragment was inserted into the plasmid PL0034 at the *Hin*dIII and *Pst*I sites upstream of the *hdhfr* cassette. Likewise, the *pbqsox* 3’ UTR fragment was inserted in the *Xho*I and *Eco*RI sites downstream of the *hdhfr* cassette to obtain the Pb-KO plasmid for *pbqsox* knockout in *P. berghei* parasites. The plasmid was linearized by *Hin*dIII/*Eco*RI digestion and transfected by electroporation into purified *P. berghei* schizonts (Janse et al., 2006). After transfection, the complete parasite suspension was injected intravenously via the tail vein into three mice. Mice were treated for 4 days with pyrimethamine (Sigma, Burlington, USA) in drinking water at 70 µg/ml starting at 24 h after injection of the transfected parasites. Three parasite clones for Pb-KO parasites were obtained by limiting dilution; integration-specific PCR was used to confirm the Pb-KO line. Primers 1 and 2 were used for diagnostic PCR of the wild-type (WT) locus, while primers 1 and 3 were used for confirming the Pb-KO line (**Supplementary Table S1**).

To generate the transgenic Pv-Tr-PbQSOX line, the *pvqsox* gene of *P. vivax* Sal-I tagged with 3×HA at its C-terminus (1662 bp + 81 bp) was synthesized (GenScript Biotech Corporation, Nanjing, China), and linked with an 858 bp upstream fragment containing the 5’ UTR of *pbqsox* gene and a 981 bp downstream fragment containing the 3’ UTR of *pbqsox* gene from *P. berghei*. Ten μg of the DNA were transfected by electroporation into purified Pb-KO schizonts, and the transfected parasites were injected intravenously via the tail vein into three mice. The Pv-Tr-PbQSOX line was selected by treating the infected mice with 5-fluorocytosine (Sigma, Burlington, USA) in drinking water (1 mg/ml in water) as described previously (Zheng et al., 2020). The correct integration of the *pvqsox* gene into the *P. berghei* Pb-KO genome was confirmed by PCR analysis using primer 1 and primer 4 (**Supplementary Table S1**).

### Pv-Tr-PbQSOX ookinete culture

For ookinete culture, mice were inoculated by intraperitoneal injection (i.p.) with 0.2 ml of infected blood containing 5×10^6^ Pv-Tr-PbQSOX parasites. Parasitemia was allowed to reach 1–3%, and mouse blood was collected on day 3 p.i. Blood was diluted 1:10 with the complete ookinete medium (RPMI 1640 containing 50 mg/l penicillin, 50 mg/l streptomycin, 100 mg/l neomycin, 20% (v/v) FBS and 1 mg/l heparin, pH 8.3) in a flask to a maximum depth of 1 cm and kept at 19°C for 24 as previously described (Sinden et al., 1985).

### Purification of Pv-Tr-PbQSOX gametocytes and ookinetes

Gametocytes were purified as previously described (Beetsma et al., 1998; Zheng et al., 2016). Pv-Tr-Pb-QSOX parasites were used to infect phenylhydrazine-treated mice. Beginning on day 4 p.i., mice were treated with 20 mg/l sulfadiazine (Sigma, Burlington, USA) in drinking water for two days. The mouse blood was collected and transferred to the pre-warmed culture medium [RPMI 1640, 20% (v/v) fetal bovine serum (FBS, Thermo), 50 mg/l penicillin and streptomycin; 50 ml/0.5ml blood] at 37°C to prevent the gametocyte activation. The blood and culture medium mixture was loaded on top of a 48% (v/v) Nycodenz/culture medium cushion, then centrifuged at 1300×g for 30 min without braking. Cells at the interface were collected and washed in RPMI 1640.

Ookinetes were cultured as described above, loaded on a 62% (v/v) Nycodenz/ookinete culture medium cushion, and purified by centrifugation at 1300×g for 30 min without braking. Ookinetes at the interface were collected and washed with RPMI 1640.

### Western blot analysis

For western blot analysis, purified Pv-Tr-PbQSOX gametocytes and ookinetes were treated with 0.15% saponin to lyse erythrocytes. Equal amounts of parasite proteins were electrophoresed on SDS-PAGE gels, transferred to a 0.22 μm PVDF membrane (Bio-Rad, Hercules, USA), and incubated with a blocking buffer (5% non-fat milk in PBS-T) for 1 h at 37°C. After blocking, the blots were probed with pooled anti-rPvQSOX rabbit immune sera (1: 1000) in the blocking buffer. After three washings with PBS-T, HRP-conjugated goat anti-rabbit IgG antibodies (1:10,000) (Invitrogen, Waltham, USA) were added as secondary antibodies. The fluorescence signals from the reaction were visualized by chemiluminescence using a Pierce ECL Western Blotting Kit (Thermo) and scanned on an Odyssey infrared imaging system (LI-COR Biosciences). The rGST immune sera were used as a negative control, and HSP70 immune sera (1: 1000) were used for protein loading control.

### Indirect immunofluorescence assay

A transgenic *P. berghei* parasite line Pv-Tr-PbQSOX expressing PvQSOX-HA was used for IFA. Parasites (gametocytes, gametes, zygotes, and ookinetes) were washed in PBS and fixed with 4% paraformaldehyde and 0.0075% glutaraldehyde (Sigma, Burlington, USA) in PBS for 30 minutes at room temperature (Tonkin et al., 2004). After permeabilization with 0.1% Triton X-100, cells were rinsed with 50 mM glycine in PBS and blocked with PBS containing 3% skim milk for 1 h at 37°C. Parasites were probed with the pooled rabbit anti-rPvQSOX sera (1:500) or control sera (1:500) in PBS containing 3% skim milk for 1 h at 37°C in a humidified chamber. All parasites were co-incubated with mouse/rabbit antisera (1: 500) against PbMSP1, P47, α-tubulin, and Pbs21 as stage-specific markers for schizonts, female gametocytes/gametes, male gametocytes/gametes, and zygote/ookinetes, respectively (Liu et al., 2019). Slides were washed 3 times with PBS for 5 min and probed with Alexa Fluor 488-conjugated α-rabbit IgG secondary antibodies (1: 500; Invitrogen, Carlsbad, CA) and Alexa-555 conjugated goat anti-mouse IgG secondary antibodies (1: 500; Cell signaling, USA) at 1:500 dilution in PBS at 37°C for 1 h. For comparison, parasites were also probed with the mouse anti-HA mAb (1:500, Invitrogen, Carlsbad, CA) using the same procedure. Parasite nuclei were stained with Hoechst 33258 (1:1,000; Invitrogen, Carlsbad, CA) and observed under a fluorescent microscope. Images were captured and processed on a Zeiss Axio Observer Z2 using Axio Vision software and Adobe Photoshop (Adobe Systems Inc.).

### Growth phenotype of the transgenic parasite

The transgenic Pv-Tr-PbQSOX and Pb-KO lines were compared to the WT *P. berghei* for their growth phenotype. BALB/c mice (5 mice/group) were inoculated i.p. with 0.2 ml of infected blood containing 5×10^6^ Pv-Tr-PbQSOX, Pb-KO, or WT *P. berghei*. Asexual parasitemia was monitored on days 3, 5, 7, 9, and 11 p.i. by examining Giemsa-stained thin blood smears. Mice were injected i.p. with 1.2 mg phenylhydrazine 3 days before infection to evaluate the gametocytogenesis and gametocyte activation. The mice were then inoculated i.p. with 5×10^6^ Pv-Tr-PbQSOX, Pb-KO, or WT *P. berghei* parasites. Gametocytemia (gametocytes per 10^4^ RBC) and female/male gametocyte ratio were counted on day 3 p.i (Lal et al., 2009). Male gametocyte exflagellation centers and the formation of ookinetes were performed and quantified as described previously (van Dijk et al., 2010; Zheng et al., 2020). The characteristic morphologies of male and female gametocytes, and exflagellation of microgametes were quantified as the images we published previously (Zheng et al., 2017). Ten μl of tail blood containing equal amounts of parasites were added to 40 μl standard ookinete medium. After induction of gamete formation at 25°C for 15 min, the culture was placed on a coverslip and analyzed under a phase-contrast microscope at 400× magnification to count the exflagellation centers in 10 min. The culture was added to another 50 μl standard ookinete medium and further incubated at 19°C for 24 h to determine the number of ookinetes. The culture was labeled with mouse anti-Pbs21 mAb (1:500) without permeabilization. The numbers of the mature ookinetes in 0.5 μl culture were counted under a fluorescence microscope (100× oil objective).

### Transmission-blocking assays

To determine whether IgGs against rPvQSOX affect male gametocyte exflagellation and the formation of ookinetes and oocysts of the transgenic parasites Pv-Tr-PbQSOX, mice were infected with Pv-Tr-PbQSOX or WT *P. berghei* parasites as described above. To determine the effect on exflagellation, purified IgGs against rPvQSOX were added to the ookinete culture at 10, 5, and 1 µg/50 µl of standard ookinete medium, respectively. After induction of gamete formation at 25°C for 15 min, exflagellation centers were counted in 10 min. For ookinetes, purified IgGs against rPvQSOX were added to the ookinete culture at 10, 5, and 1 µg/100 µl of ookinete culture, respectively. Ookinete cultures were incubated at 19°C for 24 h, and the number of ookinetes was counted.

For direct mosquito-feeding assays, mice were infected with Pv-Tr-PbQSOX or WT *P. berghei* parasites. When asexual parasitemia was approximately 5–7% after 3 days of infection, mice were injected intravenously with either 150 µg of purified anti-rPvQSOX IgGs/mouse or an equal volume of IgGs against rGST as control 1 h before mosquito feeding. At 10 days after blood feeding, midguts were dissected from ~30 *An. stephensi* mosquitoes of each group and stained with 0.5% mercurochrome to determine the prevalence and intensity of infection.

### Direct membrane feeding assays

Ten ml of venous blood was collected from four patient volunteers infected with a single species of *P. vivax* after signing informed consent at the Tha Song Yang hospital, located at the Thai-Myanmar border. Patients visiting malaria clinics at the Thai-Myanmar border were diagnosed with *P. vivax* infection by microscopy and later confirmed by nested PCR targeting 18S rRNA. All research involving human subjects in this study was reviewed and approved by the Ethics Committee of the Faculty of Tropical Medicine, Mahidol University, Bangkok, Thailand (MUTM 2018-016). Direct membrane feeding assays (DMFAs) were performed using blood from these volunteers, as previously described (Qiu et al., 2020). Giemsa-stained thin films from 1 μl blood samples were used to determine the parasite densities. Ninety μl heat-inactivated (complement minus) human AB+ serum from healthy volunteers was utilized to dilute the purified IgGs against rPvQSOX or control antibodies at 1:1 (v/v). The collected erythrocytes (180 μl) from the *P. vivax* patients were mixed with the diluted antibodies at 1:1 (v/v). The reconstituted blood samples were incubated at 37°C for 15 min and then introduced into membrane feeders. For each clinical isolate, *An. dirus* mosquitoes starved overnight before the experiment and were allowed to feed on the blood for 30 min using membrane feeders at 37°C. After several hours of rest, only fully engorged mosquitoes were maintained and fed with a daily changed cotton pad soaked with 10% sucrose solution at 27°C and 80% relative humidity for a week. Mosquito midguts were dissected and stained with 0.5% mercurochrome for 15 min and scored for oocysts with a compound microscope at 200× total magnification. In transmission-blocking assays and DMFAs, TBA was used to express the reduction in the number of infected mosquitoes, and the transmission-reducing activity (TRA) was used to evaluate the reduction in parasite load.

### Genetic polymorphisms analysis

Genomic DNA was extracted from dried filter-paper blood spots of the four *P. vivax* patients using a QIAamp DNA Blood Mini kit (Qiagen, Germany). PCR was performed to amplify the DNA fragment encoding the PvQSOX (aa 40–553) using primers PvQSOX-F (GTAGATGTGTGTAAAGGCGC) and PvQSOX-R (CTAGGAGTGTACACCTCCACG). Cycling conditions were: initial denaturing at 94 °C for 2 min, followed by 35 cycles of 94 °C for 10 s, 55 °C for 30 s, and 68 °C for 2 min, and a final extension at 68 °C for 5 min. QIAquick Gel Extraction Kit (Qiagen) was used to purify the amplified DNA products. The ABI Prism^®^ BigDye™ cycle sequencing kit was used to sequence the purified DNA.

### Statistical analyses

Statistical comparison between groups was performed with the GraphPad Prism 7.0 software. The parasitemia, gametocytemia, and female: male gametocyte ratios were used one-way analysis of variance (ANOVA) and Bonferroni’s multiple comparisons test. The exflagellation and the ookinete numbers in parasites were performed by the nonparametric Kruskal-Wallis and Dunn’s multiple-comparisons test. The intensity of infection (oocysts/midgut) was analyzed using the Mann-Whitney U test, while the prevalence of infection was analyzed using Fisher’s exact test using SPSS version 21.0. P *<* 0.05 was considered statistically significant.

## Results

### Recombinant PvQSOX protein expression and purification

**Figure 1:**
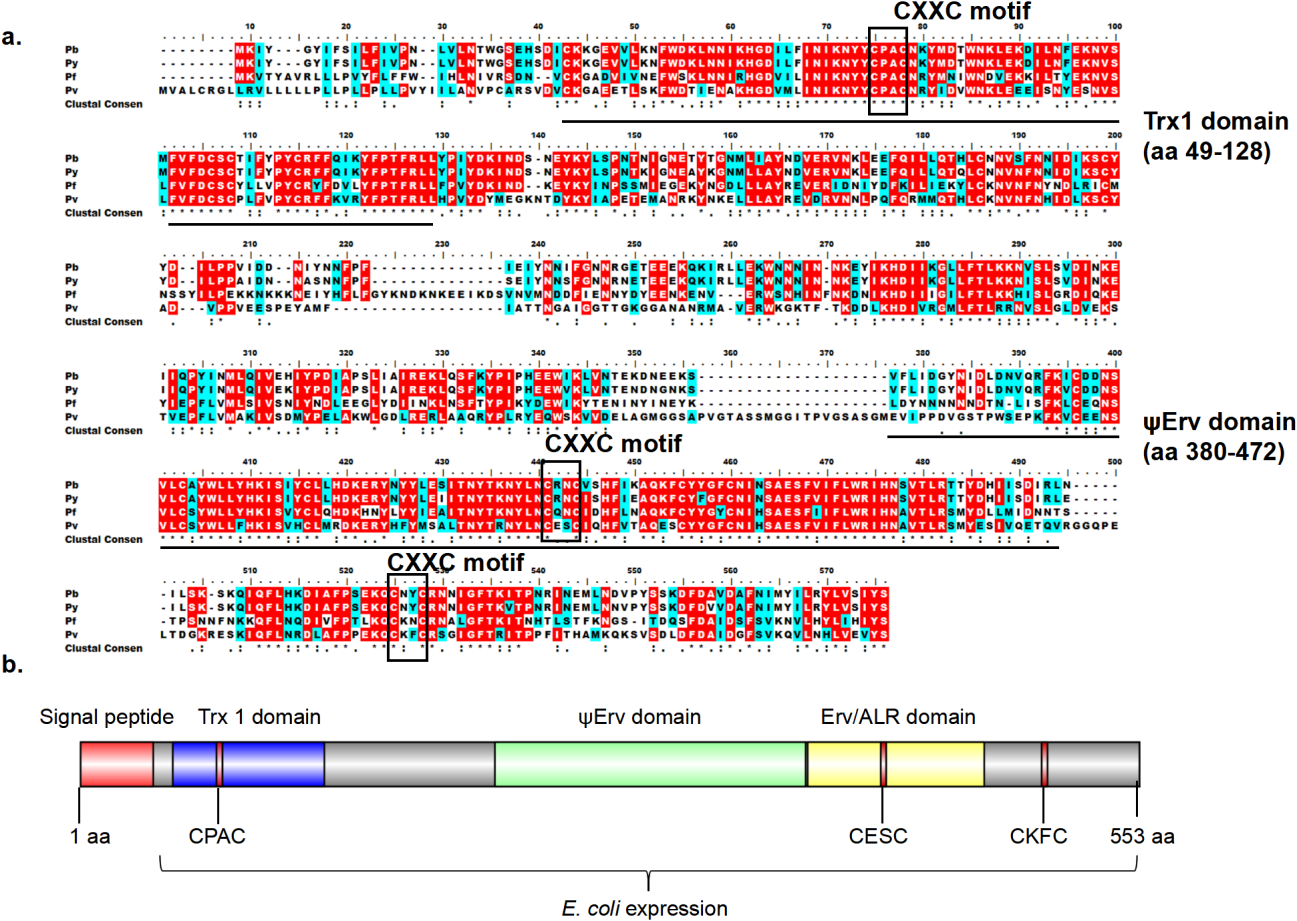
Bioinformatic analyses of PvQSOX. **(A)** ClustalW alignment of the QSOX in *P. vivax* (*Pv*), *P. berghei* (*Pb*), *P. falciparum* (*Pf*), *P. yoelii* (*Py*) showing conserved amino acids. Identical: red, similar: blue, >50% highlight. **(B)** Domain organization of PvQSOX protein. Signal peptide, Trx1, ψErv, and Erv/ALR domains are highlighted in red, blue, green, and yellow, respectively. Three CXXC motifs (Trx-CPAC, Erv-CESC, and Ct-CKFC) in PvQSOX are highlighted in red. The *Escherichia coli* expression segment shows amino acids 40 to 553.

**Figure 2:**
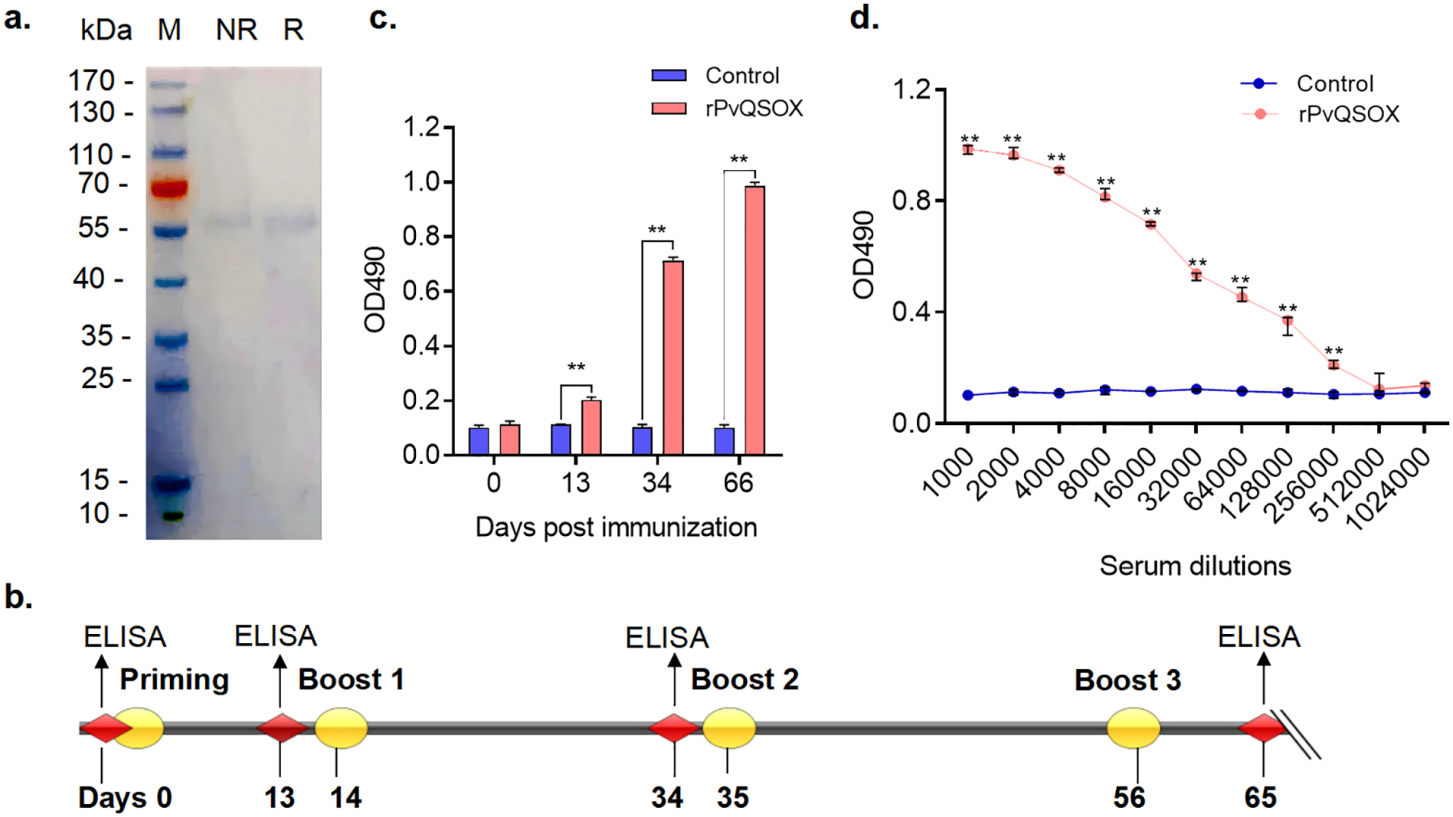
rPvQSOX proteins were purified from *Escherichia coli*, and rPvQSOX protein successfully induced the production of specific antibodies in rabbits. **(A)** Purified recombinant proteins were examined by reducing and non-reducing SDS-PAGE with Coomassie blue staining. M, molecular weight marker; NR, non-reducing; R, reducing. **(B)** Rabbit immunization and analysis scheme. **(C)** Induction of antibodies in the immunized rabbit. Rabbits were immunized with rPvQSOX or rGST, and sera samples were collected on days 0, 13, 34, and 65, then pooled for each group. The sera were diluted 1:1000 in the blocking buffer. The experiment was performed three times. The error bar shows the median with range. **p < 0.01 (Mann-Whitney *U* test, n=3). **(D)** At 10 days after the final immunization, the immune sera were pooled after the collection and then analyzed by ELISA. IgG titers correspond to the last dilution of the pooled anti-rPvQSOX sera, where OD490 values were above the cut-off values. The cut-off value was defined as that of the pooled sera from the control rabbit, OD490 + 3×SD. The experiment was performed three times. Error bar shows median with range. ** *p <* 0.01 (Mann-Whitney *U* test, n=3).

QSOX proteins are highly conserved among *Plasmodium* species (Zheng et al., 2020). PvQSOX shares 45% amino acid identity with PbQSOX (**Figure 1A**). PvQSOX has all domains typical of QSOX family proteins (**Figure 1B**). PvQSOX also has a predicted signal peptide-like PbQSOX (Zheng et al., 2020), indicating it might be a secreted protein. To express rPvQSOX, we synthesized the harmonized *pvqsox* gene without the signal peptide (1542 bp) and cloned it in the pET30a (+) vector for expression in *Escherichia coli.* After IPTG induction at 20°C for 12 h, the His-tagged rPvQSOX protein was expressed in the inclusion bodies. After denaturation, the rPvQSOX was purified using a Ni-NTA column and refolded in a refolding buffer. The rGST control protein was also expressed and purified as previously described (Qiu et al., 2020). To evaluate the formation of disulfide bonds, rPvQSOX was analyzed by reducing and non-reducing SDS-PAGE. One band with a molecular weight of approximately 59 kDa was shown under reducing conditions (**Figure 2A**), and the band run under non-reducing conditions was migrated at a slightly higher molecular weight, suggesting that intramolecular disulfide bonds were formed, as observed elsewhere (Matsusaki et al., 2019).

### Immunization with rPvQSOX induces high PvQSOX-specific antibody titers

To assess the immunogenicity of rPvQSOX protein, we vaccinated rabbits with rPvQSOX and tested the resultant sera by ELISA. The immunization and analysis scheme is shown in **Figure 2B**. After the first immunization, compared with the control sera, rabbits developed rPvQSOX-specific antibodies, and titers significantly increased in subsequent booster immunizations at 34 and 65 days (*p <* 0.01, **Figure 2C**). Specifically, compared with the control sera, the rPvQSOX-specific antibody titer increased to 1: 256,000 after the third booster (*p <* 0.01, **Figure 2D**). We used the same immunization scheme for the control rGST protein (Qiu et al., 2020).

### Generation of a transgenic *P. berghei* parasite (Pv-Tr-PbQSOX)

**Figure 3:**
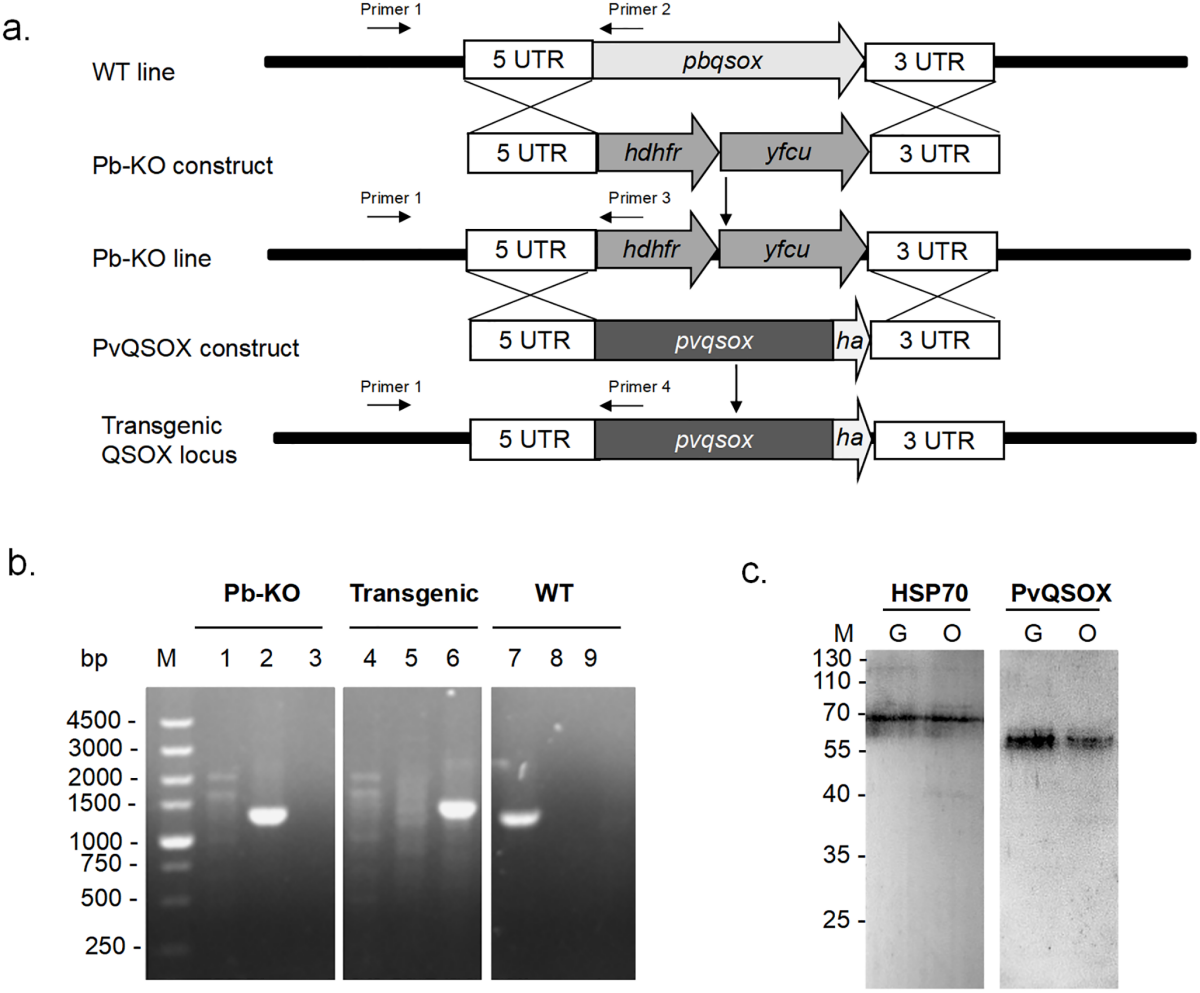
Generation of Pb-KO and Pv-Tr-PbQSOX parasite line. **(A)** 5’ UTR and 3’ UTR sequences of the *pbqsox* gene were used to target the genomic locus in *P. berghei* by double-crossover recombination. The plasmid used for to Pb-KO construct contained a cassette of *hdhfr* and *yfcu* gene flanked by 5’ UTR and 3’ UTR of *pbqsox*. The *hdhfr* and *yfcu* gene was used for drug selection. The full-length *pvqsox* sequence tagged with 3 ×HA at its C-terminus by flanking the 5’ UTR and 3’ UTR fragments of the *pbqsox* gene was used to target the Pb-KO line by double-crossover recombination. Primers 1 + 2 were used for diagnostic PCR of the WT locus, primers 1 + 3 were used to confirm Pb-KO of *pbqsox*, while primers 1 + 4 were used to confirm PvQSOX-expressing transgenic parasite Pv-Tr-PbQSOX (Transgenic). **(B)** PCR verification of Pb-KO and Pv-Tr-PbQSOX. Lanes 1, 2, and 3 were Pb-KO parasites; lanes 4, 5, and 6 were Pv-Tr-PbQSOX transgenic parasites; while lanes 7, 8, and 9 were WT parasites. Lanes 1, 4, and 7, PCR with primers 1 + 2 (1378 bp); lanes 2, 5, and 8, PCR with primers 1 + 3 (1333 bp); lanes 3, 6, and 9, PCR with primers 1 + 4 (1442 bp). **(C)** Western blot of PvQSOX in sexual-stage parasites. Ten μg parasite antigens of gametocytes (G) or ookinetes (O) per line were incubated with anti-rPvQSOX sera (1:1000) from immunized rabbits. Protein loading is estimated using HSP70 immune sera (1:1000).

We first used a double-crossover recombination strategy to produce a Pb-KO line, where the *pbqsox* gene was deleted (**Figure 3A**). Selected pyrimethamine-resistant parasites were cloned for genotype analyses, and *pbqsox* deletion was confirmed by integration-specific PCR (**Figure 3A**). As expected, a PCR product of 1,333 bp was detected in the Pb-KO parasite but not in the WT parasite (**Figure 3B**). Subsequently, the drug cassettes in the Pb-KO parasite were replaced with the coding region of PvQSOX using the same strategy. The resultant transgenic parasite line Pv-Tr-PbQSOX was further selected by negative selection with 5-fluorocytosine. Integration-specific PCR with primers 1 and 4 confirmed the generation of the Pv-Tr-PbQSOX parasite (**Figure 3B**). For comparison, PCR with primers 1 and 2 only detected a 1,378 bp product in the WT parasites (**Figure 3B**). The expression of PvQSOX proteins in the transgenic parasites was further verified by western blot analysis using the rabbit anti-rPvQSOX sera. A protein band of approximately 63 kDa was observed in Pv-Tr-PbQSOX gametocytes and ookinetes (**Figure 3C**), whereas it was not observed with the control anti-rGST sera (data not shown).

### Growth phenotype of the Pv-Tr-PbQSOX line

**Figure 4:**
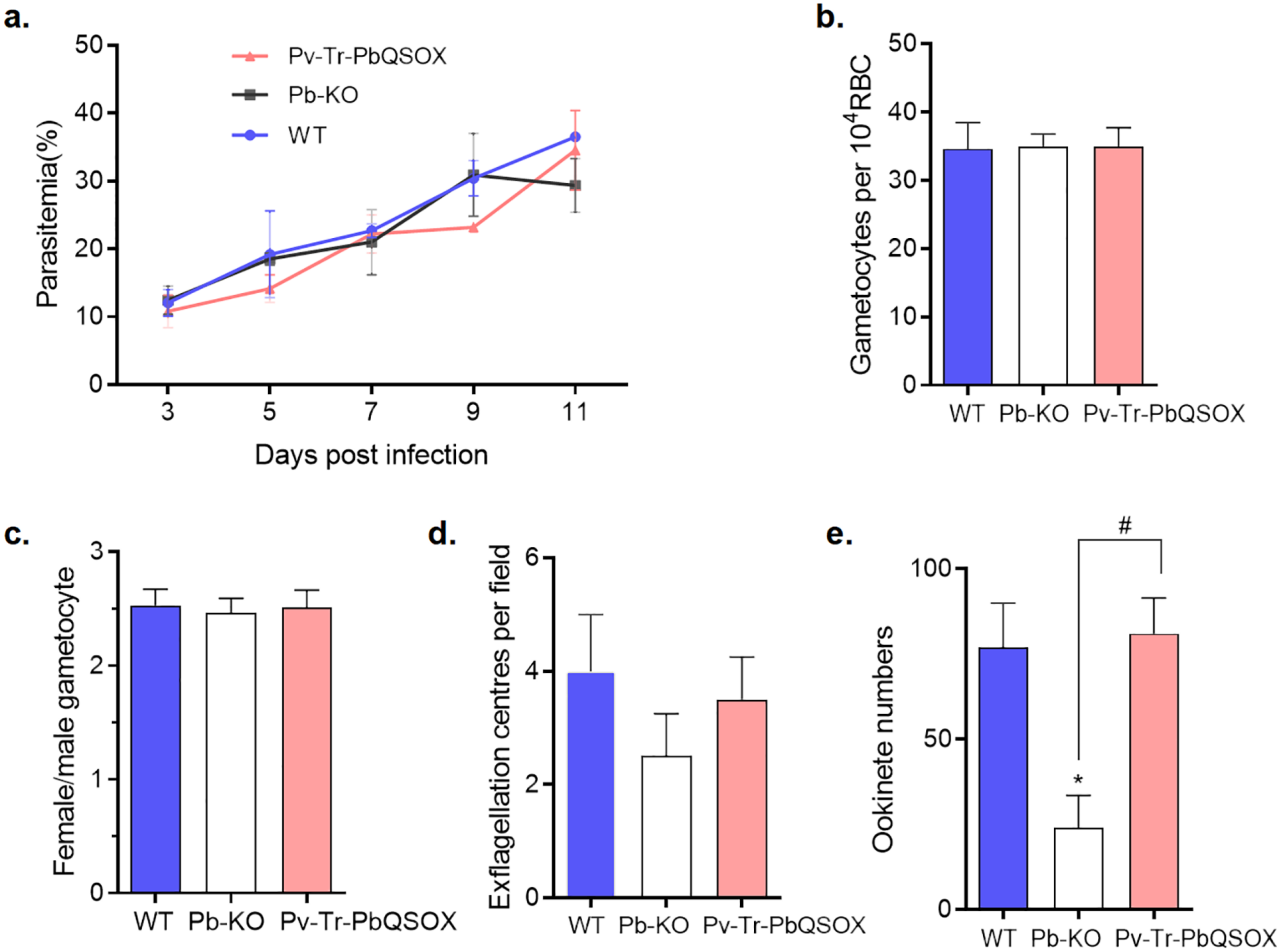
Growth phenotypes of Pv-Tr-PbQSOX. **(A)** Mice were infected with Pv-Tr-PbQSOX, Pb-KO, or WT parasites. Parasitemia was monitored for 3, 5, 7, 9, and 11 days. **(B)** Gametocytemias in mice infected with Pv-Tr-PbQSOX, Pb-KO, or WT parasites. **(C)** Female: male gametocyte ratios of Pv-Tr-PbQSOX, Pb-KO, or WT parasites. **(D)** Exflagellation of Pv-Tr-PbQSOX, Pb-KO, or WT microgametocytes. **(E)** Ookinete numbers in Pv-Tr-PbQSOX, Pb-KO, or WT parasites. In **(A–E)**, the experiment was performed three times. Statistical significance of a-c was analyzed by one-way analysis of variance (ANOVA) and Bonferroni’s multiple comparisons test, the error bars indicate mean ± SEM (n = 5). The statistical significance of d-e was analyzed by the nonparametric Kruskal-Wallis and Dunn’s multiple-comparisons test, and the error bars indicate median with IQR (n = 5).* *p <* 0.05 indicates the statistical significance between WT and Pb-KO groups. # *p <* 0.05 indicates the statistical significance between Pb-KO and Pv-Tr-PbQSOX groups.

Since PvQSOX only showed 45% amino acid identity to PbQSOX, we next examined whether the replacement of *pbqsox* by *pvqsox* affected the parasite’s life cycle. As expected from the expression patterns of QSOX, the deletion of *pbqsox* or replacement of *pbqsox* with *pvqsox* did not affect the asexual growth of the parasite (**Figure 4A**). In addition, the gametocytemia and gametocyte sex ratio were similar among the Pv-Tr-PbQSOX, Pb-KO, and WT parasites (**Figures 4B, C**). However, compared to the WT parasites, knockout of *pbqsox* led to a 33.3% reduction in exflagellation centers (**Figure 4D**) and a 68.6% reduction in ookinete numbers (*p <* 0.05; **Figure 4E**), consistent with our earlier results (Zheng et al., 2020). Remarkably, the replacement of *pbqsox* with *pvqsox* completely rescued the defects of Pb-KO in exflagellation and ookinete formation (**Figure 4E**), indicating the functional equivalence of PvQSOX in *P. berghei*.

### PvQSOX expression and localization transgenic parasites

**Figure 5:**
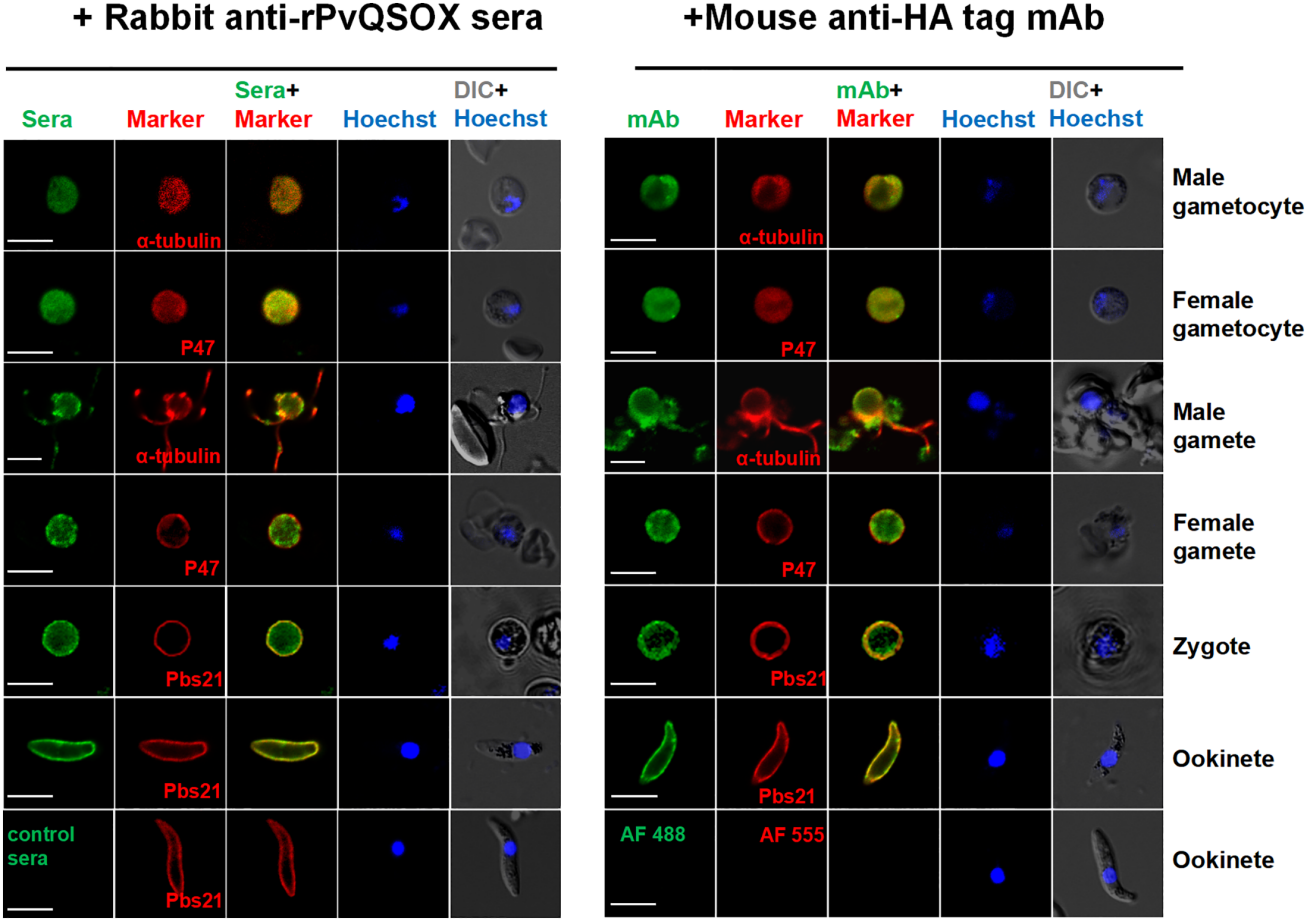
Expression and localization of PvQSOX. Immunofluorescence assays of Pv-Tr-PbQSOX different developmental stage parasites using the pooled anti-rPvQSOX rabbit immune sera (1: 500, left) or mouse anti-HA tag mAb (1:500, right) (green). Cells were permeabilized with 0.1% Triton X-100. The mouse (left)/rabbit (right) antisera (1: 500) against a-tubulin for male gametocytes/gametes, P47 for female gametocytes/gametes, and Pbs21 for ookinetes were used as stage-specific markers (red). Nuclei were stained with Hoechst (blue). The right panels are the differential interference contrast images (DIC) of the samples in the respective left panels. WT ookinetes labeled control sera or probed only with the secondary antibodies (AF 488: Alexa Fluor 488; and AF 555: Alexa Fluor 555) were used as a negative control. Scale bars = 5 µm.

Next, we verified the expression pattern of the PvQSOX protein in the transgenic parasite Pv-Tr-PbQSOX by IFA using the rabbit anti-rPvQSOX sera and the mouse anti-HA mAb. Specific fluorescence signals were detected in gametocytes, gametes, zygotes, and ookinetes (**Figure 5**). In macrogametes, zygotes, and ookinetes, strong fluorescence was observed at the periphery of the parasites, indicating the association of PvQSOX with the plasmic membrane in these stages. In contrast, asexual blood-stage parasites showed no fluorescent signals (**Supplementary Figure S1**).

### TRA and TBA of anti-rPvQSOX IgGs for transgenic parasite

**Figure 6:**
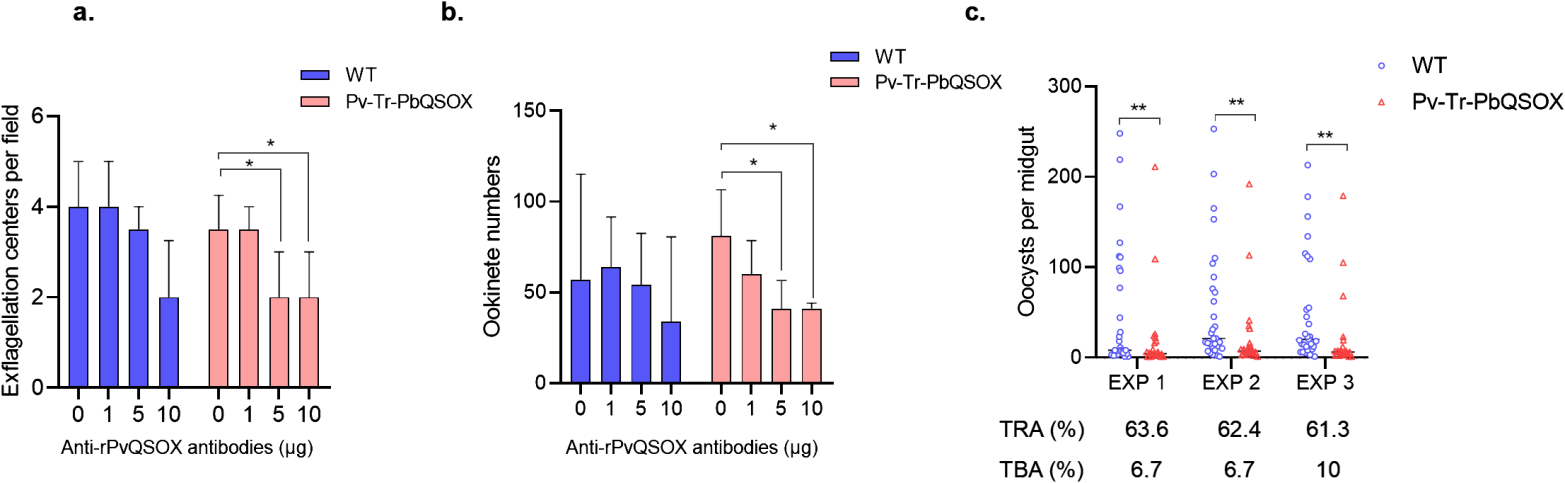
TB Activities of anti-rPvQSOX IgGs to transgenic parasites. **(A)** Effect of anti-rPvQSOX IgGs on exflagellation of male gametocytes. Exflagellation centers per field of WT *P. berghei* line and transgenic parasites Pv-Tr-PbQSOX were measured after 15 min incubation with anti-rPvQSOX IgGs. **(B)** Effect of anti-rPvQSOX IgGs on *P. berghei* ookinete formation *in vitro*. In a and b: anti-rPvQSOX IgGs were added at 0, 1, 5, and 10 μg/100 μl in cultures. The experiments were performed three times. Continuous data in groups a-b were expressed as median and IQR. The Kruskal-Wallis test was initially used for data comparison due to the skewed distribution of the data. Dunn’s multiple comparisons test was used to compare the 0 μg group with the 1μg, 5 μg, or 10 μg groups. *p < 0.05 indicates the statistical significance. **(C)** Passive antibody transfer experiment to assess the TB activity of the anti-rPvQSOX IgGs to transgenic parasites. For each isolate, about 30 *An. stephensi* mosquitoes were treated. Statistical significance of c was analyzed by the Mann-Whitney *U* test and the horizontal bars indicate the median number of oocysts per midgut. ** *p <* 0.01 indicates the statistical significance between WT and Pv-Tr-PbQSOX groups. The TRA and TBA are shown in **(C)**.

We examined the TRA (evaluate the reduction in parasite load) and TBA (evaluate the reduction in the number of infected mosquitoes) of purified anti-rPvQSOX IgGs using the WT *P. berghei* line and transgenic Pv-Tr-PbQSOX line. Using *in vitro* culture, we evaluated the effect of the antibodies on exflagellation and ookinete formation. At 1 µg/100 µl, the anti-rPvQSOX IgGs did not affect exflagellation in both WT and Pv-Tr-PbQSOX lines (**Figure 6A**). The addition of anti-rPvQSOX IgGs to 5 and 10 µg/100 µl resulted in a slight decrease in the number of exflagellation centers in WT parasites, but this was not statistically significant. This phenomenon may be attributed to PvQSOX showing 45% amino acid identity to PbQSOX, anti-rPvQSOX antibodies have a certain influence in exflagellation and ookinete formation of the WT *P. berghei* line. However, compared with the control group (0 µg/100 µl), anti-rPvQSOX IgGs at 5 and 10 µg/100 µl reduced the number of exflagellation centers in the Pv-Tr-PbQSOX line by 36.3% (*p <* 0.05; **Figure 6A**) and 41.2% (*p <* 0.05; **Figure 6A**), respectively. A similar effect of the antisera was observed in ookinete formation. Ookinete cultures with anti-rPvQSOX IgGs added at 5 and 10 µg/100 µl reduced the number of ookinetes of the Pv-Tr-PbQSOX line by 44.0% (*p <* 0.05; **Figure 6B**) and 51.6% (*p <* 0.05; **Figure 6B**), respectively. Next, we evaluated the TB effect of the anti-rPvQSOX IgGs in an antibody transfer experiment, where mice infected with WT *P. berghei* or Pv-Tr-PbQSOX strains were injected intravenously with anti-rPvQSOX IgGs 1 h before mosquito feeding. Compared with WT *P. berghei*, mosquitoes feeding on Pv-Tr-PbQSOX infected mice showed a 63.6%, 62.4%, and 61.5% reduction in oocyst density (**Figure 6C**; **Table 1**; *p <* 0.01), respectively. Compared to the WT line, the anti-rPvQSOX IgGs reduced infection prevalence by 6.9%, 6.7%, and 10.3% (**Figure 6C**; **Table 1**), respectively, while the effect was insignificant.

**Table 1:** Transmission-blocking effect of Anti-rPvQSOX IgGs for transgenic parasites.

Experiment	Group	Oocyst number Median (IQR)	Mean oocysts number	TRA (%) ^a^	*p* value ^b^	Infection rate (%)(Inf/Diss) ^c^	TBA (%) ^d^	*p* value ^e^
Exp 1	WT	8.0 (3.75-96.75)	50.1	63.6	0.009^**^	96.7 (29/30)	6.9	0.612
	Pv-Tr-PbQSOX	4.0 (1.0-10.75)	18.2			90.0 (27/30)		
Exp 2	WT	21.0 (11.5-79.25)	53.5	62.4	0.001^**^	100.0 (30/30)	6.7	0.492
	Pv-Tr-PbQSOX	6.0 (3.0-11.25)	20.1			93.3 (28/30)		
Exp 3	WT	19.5 (8.75-68.5)	49.7	61.5	0.000^**^	96.7 (29/30)	10.3	0.353
	Pv-Tr-PbQSOX	5.0 (2.0-7.25)	19.1			86.7 (26/30)		

^a^ TRA was calculated as (mean oocyst density _WT_ – mean oocyst density _Pv-Tr-PbQSOX_/ mean oocyst density _WT)_ × 100%.

^b^ Mean number of oocyst was statistically analyzed (Mann–Whitney *U* test), and *P*-values less than 0.05 were considered statistically significant.

^c^ The infection prevalence was calculated by the number of oocyst-infected mosquitoes per 30 mosquitoes dissected in each group (Inf/Diss).

^d^ TBA was calculated as (% prevalence _WT_– % prevalence _Pv-Tr-PbQSOX_/ %prevalence _WT_ ) × 100%.

^e^ Prevalence was statistically analyzed by Fisher’s exact test.

^**^ *p* < 0.01.

### TRA and TBA of anti-rPvQSOX IgGs for clinical *P. vivax* isolates

**Figure 7:**
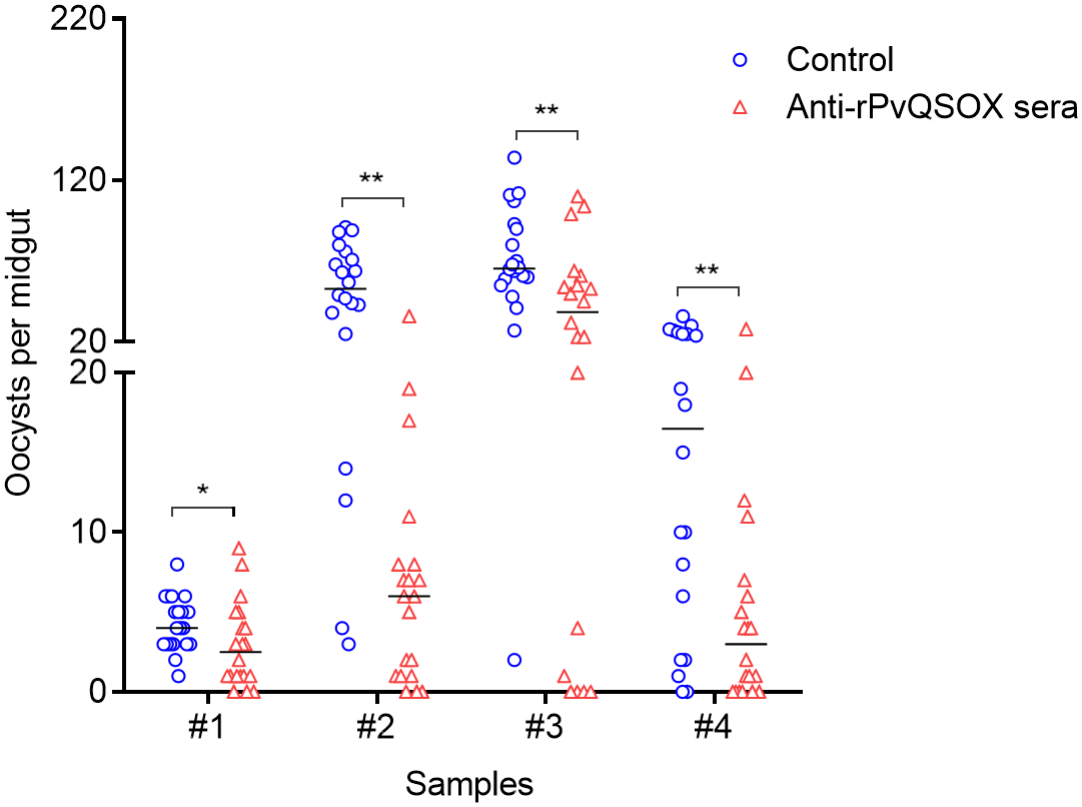
Transmission-blocking assays of rabbit antisera produced by rPvQSOX. DMFA was carried out using four *P. vivax* isolates with rabbit IgGs [anti-rPvQSOX rabbit IgGs, or anti-rGST control IgGs] mixed with unheated human serum. For each isolate, about 20 *An. dirus* mosquitoes were treated. Individual data points represent the number of oocysts/midgut. Statistical significance was analyzed by the Mann-Whitney *U* test and the horizontal bars indicate the median number of oocysts per midgut. * *p <* 0.05, ** *p <* 0.01 indicates the statistical significance between WT and Pv-Tr-PbQSOX groups.

To examine the TRA and TBA of rabbit anti-rPvQSOX antibodies for *P. vivax*, DMFA was carried out using *P. vivax* clinical isolates from four *P. vivax* patients. In all cases, the anti-rPvQSOX IgGs showed significant TRA, although the levels of oocyst reduction varied with different patient isolates. *An. dirus* mosquitoes feeding on four blood isolates mixed with the anti-rGST antibodies in DFMA had mean midgut oocyst intensity of 4.25, 51.30, 70.65, and 15.60, respectively. In comparison, mosquitoes feeding on the blood isolates mixed with the anti-rPvQSOX IgGs showed a mean infection intensity of 2.90, 7.20, 39.90, and 5.30 oocysts/midgut, corresponding to 32.00, 85.96, 43.52, and 66.03% reductions in oocyst density, respectively (**Figure 7**; **Table 2**). Compared to the control antibodies, the anti-rPvQSOX IgGs also showed a modest reduction of infection prevalence by 15, 15, 20, and 22.22%, respectively (**Figure 7**; **Table 2**), while the effect was insignificant.

**Table 2:** Transmission-blocking effect of anti-rPvQSOX sera for *P. vivax* samples collected in Thailand.

*P.vivax* isolates	Group	Oocyst number Median (IQR) ^a^	Mean oocysts number	TRA (%) ^b^	*p* value^c^	Infection rate (%) ^d^(Inf/Diss)	TBA (%) ^e^	*p* value ^f^
Isolate #1	Control	4.0 (3.0-5.75)	4.25			100 (20/20)		
	PvQSOX	2.5 (1.0-4.75)	2.90	32.00	0.0301^*^	85 (17/20)	15.00	0.2308
Isolate #2	Control	53.0 (28.25-74.75)	51.30			100 (20/20)		
	PvQSOX	6.0 (1.0-8.0)	7.20	85.96	0.0001^**^	85 (17/20)	15.00	0.2308
Isolate #3	Control	65.5 (56.0-92.25)	70.65			100 (20/20)		
	PvQSOX	38.5 (1.75-59.5)	39.90	43.52	0.0024^**^	80 (16/20)	20.00	0.1060
Isolate #4	Control	16.5 (3.0-25.75)	15.60			90 (18/20)		
	PvQSOX	3.0 (0.0-6.75)	5.30	66.03	0.0042^**^	70 (14/20)	22.22	0.2351

^a^ IQR: inter-quartile range.

^b^ TRA was calculated as (mean oocyst density _WT_ – mean oocyst density _Pv-Tr-PbQSOX_/ mean oocyst density _WT)_ × 100%.

^c^ Mean number of oocyst was statistically analyzed (Mann–Whitney *U* test), and *P*-values less than 0.05 were considered statistically significant.

^d^ The infection prevalence was calculated by the number of oocyst-infected mosquitoes per 20 mosquitoes dissected in each group (Inf/Diss).

^e^ TBA was calculated as (% prevalence _WT_– % prevalence _Pv-Tr-PbQSOX_/ %prevalence _WT_ ) × 100%.

^f^ Prevalence was statistically analyzed by Fisher’s exact test.

^*^ *p* < 0.05, ^**^ *p* < 0.01.

To determine whether the variation of TRA among these four isolates used in DMFA might be attributed to genetic polymorphisms of the *pvqsox* gene, we sequenced the DNA fragment of *pvqsox* in the four clinical *P. vivax* isolates. We evaluated the genetic polymorphism of PvQSOX (aa 40–553) in the four clinical *P. vivax* isolates. The isolate #1 sequence was the same as the *P. vivax* Sal-I sequence, whereas isolates #2, #3, and #4 all had the M361I substitution.

## Discussion

In our screening for TBV candidates using the rodent malaria model *P. berghei*, we identified PbQSOX as a promising transmission-blocking target, which can induce antibodies in mice with considerable activities in inhibiting *P. berghei* ookinete conversion *in vitro* and oocyst development in mosquito-feeding assays (Zheng et al., 2020). This study investigated the transmission-blocking potential of the PvQSOX, the ortholog of QSOX in *P. vivax*, using *in vivo* studies with a transgenic *P. berghei* parasite line expressing PvQSOX and *in vitro* with *P. vivax* clinical isolates. We demonstrated the immunogenicity of the recombinant PvQSOX protein expressed in *Escherichia coli* and showed that the anti-PvQSOX antibodies raised in rabbits possessed significant levels of TRA using both *in vivo* and *in vitro* systems.

QSOX is a specialized enzyme that catalyzes the direct introduction of disulfide bonds into unfolded, reduced proteins and participates in the protein folding process (Turturice et al., 2013). As a member of the thioredoxin superfamily, PbQSOX possibly regulates the redox state and maintains the integrity of ookinete surface molecules. It plays an essential role in the sexual development of *P. berghei*, since *pbqsox* deletion substantially reduces the exflagellation of male gametocytes and the infectivity of the Pb-KO parasite to mosquitoes (Zheng et al., 2020). QSOX is highly conserved among *Plasmodium* species; PvQSOX displays 45% amino acid identity to PbQSOX and contains all conserved protein domains characteristic of QSOX family proteins, suggesting PvQSOX likely has similar enzyme activities. The finding that PvQSOX fully rescued the defects in sexual development of the Pb-KO parasite line confirmed the functional conservation of QSOX in *Plasmodium*.

Transgenic rodent malaria parasites expressing human malaria antigens (e.g., PfCSP, PvCSP, Pfs25, Pvs25, Pvs48/45) have been developed over the past decade; most were used as surrogates to evaluate the immunogenicity and protective efficacy of vaccines (Ramjanee et al., 2007; Goodman et al., 2011; Iyori et al., 2013; Salman et al., 2017; Cao et al., 2018). The lack of a long-term *in vitro* culture of *P. vivax* further emphasizes the significance of the transgenic parasites for vaccine development. In this study, we generated a transgenic *P. berghei* parasite line Pv-Tr-PbQSOX, where the coding region of PbQSOX was replaced with that of PvQSOX, and found that the transgenic parasite and the WT parasite had similar asexual growth and sexual development (Zheng et al., 2020). PvQSOX also showed a similar expression and localization pattern as PbQSOX, with the notable association of QSOX proteins with the plasma membrane of zygotes and ookinetes (Zheng et al., 2020). The protein expression and localization features served as the design principle of the malaria TBVs. Using this transgenic parasite line, we evaluated *in vitro* and *in vivo* TRA of PvQSOX and found that the anti-PvQSOX IgGs reduced exflagellation and ookinete conversion *in vitro*. In an antibody transfer experiment, the anti-rPvQSOX IgGs reduced oocyst density by 63.6%, 62.4%, and 61.5% in mosquitoes. These results extended the transmission-blocking potential of PvQSOX and illustrated the usefulness of the transgenic rodent parasite for evaluating *P. vivax* vaccine candidates.

Standard and direct membrane-feeding assays (SMFA and DMFA) are the most epidemiologically relevant methods to evaluate the TRA and TBA of vaccine candidates (Miura et al., 2020). DMFA using the clinical *P. vivax* isolates not only allows the better assessment of TRA of antibodies elicited by vaccination but also allows the prediction of the protective potential of these antibodies if tested in clinical trials in endemic areas (McCaffery et al., 2019). Therefore, we further evaluated the TRA and TBA of the anti-rPvQSOX antibodies using DMFA with four clinical *P. vivax* isolates. We found that the purified anti-rPvQSOX IgGs reduced oocyst density by 32–86% and infection prevalence by 15–20%. This variation is intrinsic to the DMFA method, given that the variable gametocyte density in the blood affects the infection rate of mosquitoes (Churcher et al., 2012; Bompard et al., 2017). In this case, the variation may be unrelated to the genetic polymorphism in PvQSOX.

Both in an antibody transfer experiment using transgenic *P. berghei* parasite line Pv-Tr-PbQSOX and in DMFA, the TRA, and TBA did not reach 100%, partial parasites can still transmit. Given that the antigen-induced rabbit antibodies have only moderate activity, it is valuable to know whether this could be (partially) explained by the quality of the antigen. In the SDS-PAGE analysis, it looks like the bands under reducing and non-reducing conditions run at (slightly) different heights, suggesting that intramolecular disulfide bonds were formed. However, the rPvQSOX protein was expressed in the inclusion bodies in the *Escherichia coli* expression system, the process of protein purification undergoes denaturation and renaturation. More confirmatory experiments need to be used to determine the quality of the antigen in terms of intramolecular disulfide bonds, proper folding and oligomeric state, etc, and its effect on antibody titer. On the other hand, knockout *pbqsox* in *P. berghei* can still infect mosquitoes (Zheng et al., 2020), which reminds us that PvQSOX alone as a transmission-blocking vaccine candidate may have limitations. Multiple measures should be found to improve the transmission-blocking effect, such as combining some effective *Plasmodium* sexual surface antigens to explore the TRA and TBA may be meaningful.

It is noteworthy there are several limitations to our study. First, as our goal was on the TBV potential of PvQSOX, we did not analyze the entire life cycle of the transgenic rodent parasites, so the development of the oocysts and sporozoites of Pv-Tr-PbQSOX was not clear. Second, in our study, Freund’s adjuvant was used to immunize rabbits in this study. However, Freund’s adjuvant is not suitable for human use, the optimized adjuvants suitable for clinical trials deserve future evaluations. Third, the antigenic epitopes of PvQSOX need to be mapped and assessed for their incorporation into chimeric vaccine designs targeting multiple antigens.

## Data Availability

The datasets presented in this study can be found in online repositories. The names of the repository/repositories and accession number(s) can be found in the article/**Supplementary Material**.

## Supplementary material

Supplementary Table 1 Primer information and sequences.

Supplementary Figure 1 Immunofluorescence assays of Pv-Tr-PbQSOX different developmental stage parasites using the pooled anti-rPvQSOX rabbit immune sera (1:500) or mouse anti-HA tag mAb (1:500) (green). Cells were permeabilized with 0.1% Triton X-100. The mouse (up)/rabbit (down) antisera (1: 500) against PbMSP1 for schizonts were used as stage-specific markers (red). Nuclei were stained with Hoechst (1:1000) (blue). The right panels are the differential interference contrast images (DIC) of the samples in the respective left panels. Scale bars = 5 µm.
