# Supplementary material for: Immunogenicity and transmission-blocking potential of quiescin sulfhydryl oxidase in Plasmodium vivax
Source: Front Cell Infect Microbiol. 2024 Aug 27;14:1451063. doi: 10.3389/fcimb.2024.1451063 (PMC11385281; doi:10.3389/fcimb.2024.1451063)
Supplement: Supplementary Table 1 — Primer information and sequences. [file Table1.docx]

**Table S1 Primer information and sequences.**

| PbQSOX KO plasmid | 5UTR-F | CCCAAGCTTTTGTTTGGTCTCGCTG |
| --- | --- | --- |
|  | 5UTR-R | AAAACTGCAGTATTTTTCTTTAATATTTTATTAAACAGATAAAT |
|  | 3UTR-F | CCGCTCGAGAGTTTTTTCCTAACATCGATG |
|  | 3UTR-R | CCGGAATTCCAATGACGCTTCTAACGATTTAAATA |
| KO line and Pv-Tr-PbQSOX integration-specific PCR | Primer 1 | TATGAAAACGCTAATACTAGTCCA |
|  | Primer 2 | TGAATGTTCCGAACCCCA |
|  | Primer 3 | GGTGCTTTGAGGGGTGAG |
|  | Primer 4 | GCGTTTTCTATGGTGTCCCA |
