# Supplementary figures and images for: Immunogenicity and transmission-blocking potential of quiescin sulfhydryl oxidase in Plasmodium vivax
Source: Front Cell Infect Microbiol. 2024 Aug 27;14:1451063. doi: 10.3389/fcimb.2024.1451063 (PMC11385281; doi:10.3389/fcimb.2024.1451063)

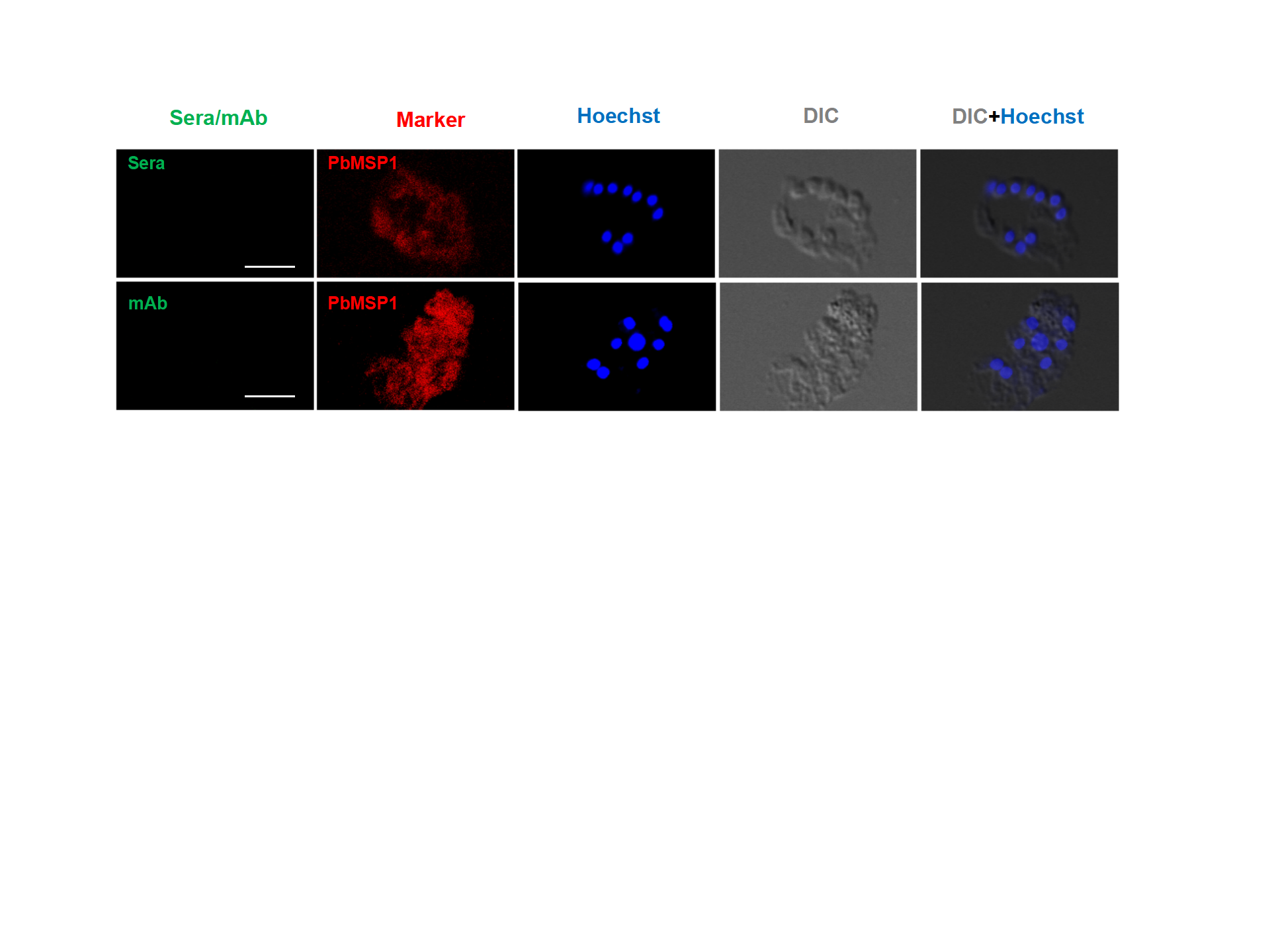

Supplement: Supplementary Figure 1 — Immunofluorescence assays of Pv-Tr-PbQSOX different developmental stage parasites using the pooled anti-rPvQSOX rabbit immune sera (1:500) or mouse anti-HA tag mAb (1:500) (green). Cells were permeabilized with 0.1% Triton X-100. The mouse (up)/rabbit (down) antisera (1: 500) against PbMSP1 for schizonts were used as stage-specific markers (red). Nuclei were stained with Hoechst (1:1000) (blue). The right panels are the differential interference contrast images (DIC) of the samples in the respective left panels. Scale bars = 5 µm. [file Image1.tif]
